# Metalloprotein catalysis: structural and mechanistic insights into oxidoreductases from neutron protein crystallography

**DOI:** 10.1107/S2059798321009025

**Published:** 2021-09-27

**Authors:** Gabriela C. Schröder, Flora Meilleur

**Affiliations:** a Department of Molecular and Structural Biochemistry, North Carolina State University, Raleigh, NC 27695, USA; b Neutron Scattering Division, Oak Ridge National Laboratory, Oak Ridge, TN 37831, USA

**Keywords:** neutron protein crystallography, X-ray diffraction, metalloproteins, enzymatic mechanisms, protonation, radiation damage

## Abstract

Metalloproteins catalyze a range of reactions, with enhanced chemical functionality due to their metal cofactor. The reaction mechanisms of metalloproteins have been experimentally characterized by spectroscopy, macromolecular crystallography and cryo-electron microscopy. An important caveat in structural studies of metalloproteins remains the artefacts that can be introduced by radiation damage. Photoreduction, radiolysis and ionization deriving from the electromagnetic beam used to probe the structure complicate structural and mechanistic interpretation. Neutron protein diffraction remains the only structural probe that leaves protein samples devoid of radiation damage, even when data are collected at room temperature. Additionally, neutron protein crystallography provides information on the positions of light atoms such as hydrogen and deuterium, allowing the characterization of protonation states and hydrogen-bonding networks. Neutron protein crystallo­graphy has further been used in conjunction with experimental and computational techniques to gain insight into the structures and reaction mechanisms of several transition-state metal oxidoreductases with iron, copper and manganese cofactors. Here, the contribution of neutron protein crystallo­graphy towards elucidating the reaction mechanism of metalloproteins is reviewed.

## Abstract 2

Neutron protein crystallography provides insight into the structure and reaction mechanism of transition-state metal oxidoreductases without resulting in radiation-damage-induced artefacts.

## 1. Introduction   

Metals play a central role in biology, particularly in association with the proteome, where they have catalytic, electron-transfer, structural and storage roles (Holm *et al.*, 1996 ▸). Metal cofactors provide proteins with an enriched functional range by expanding their physiochemical properties and playing a role in activation and stabilization during catalysis (Hemschemeier & Happe, 2018 ▸; Nastri *et al.*, 2019 ▸). The reaction mechanism of metalloproteins has been explored using a plethora of techniques including structural, spectroscopic and computational studies (Fontecilla-Camps & Nicolet, 2014 ▸). To gain a more complete understanding of protein interactions and catalysis, a complete, all-atom structure is a requisite. Neutron protein crystallography remains the sole structural technique that can determine H-atom positions without radiation-induced damage both at room temperature and under cryo-conditions, making it particularly valuable for the study of metallo­proteins and their reaction mechanisms (Bodenheimer *et al.*, 2017 ▸; Meilleur *et al.*, 2018 ▸, 2020 ▸; Ashkar *et al.*, 2018 ▸).

### 1.1. Metals in biology   

Metalloproteins make up approximately one third of the structures deposited in the Protein Data Bank (PDB; Putignano *et al.*, 2018 ▸). Within metalloenzymes, metal cofactors are coordinated to protein residues in the primary coordination sphere, where liganded amino acids finely tune the metal reactivity and redox potentials for optimal catalysis (Riordan, 1977 ▸; Hosseinzadeh & Lu, 2016 ▸). The chemistry afforded by the metal cofactor is determined primarily by the identity of the metal; however, the secondary coordination sphere has a considerable influence on enzyme reactivity (Maglio *et al.*, 2012 ▸). The secondary coordination sphere is composed of the residues that are proximal but not directly bonded to the metal ion and plays an important role in reactivity, while also ensuring substrate specificity and regio­selectivity (Colquhoun *et al.*, 1986 ▸). Of particular interest are metal-cofactor-containing oxidoreductases that are involved in the critical processes of electron exchange between donor and acceptor molecules in reactions as varied as oxygen activation and insertion, electron transfer, hydride transfer and hydrogen abstraction (Younus, 2019 ▸). Oxidoreductases make up approximately 44% of the Enzyme Commission classes (EC class 1) and are coordinated to a range of metals including iron, copper, manganese, nickel and zinc (Andreini *et al.*, 2008 ▸). Gaining a detailed structural understanding of the primary and secondary coordination spheres is central to elucidating the reaction mechanism of these metalloproteins, which will help to inform improvements for pharmaceutical and biotechnological applications (Bowman *et al.*, 2016 ▸).

### 1.2. Structural studies and radiation damage   

An essential aspect of elucidating the reaction mechanism of metalloproteins is the ability to visualize the positions of H atoms in amino-acid residues, water molecules and reaction intermediates, since H atoms are central to enzyme chemistry (Engler *et al.*, 2003 ▸; Hubbard & Kamran Haider, 2010 ▸; Bowie, 2011 ▸; Woińska *et al.*, 2016 ▸; Clabbers *et al.*, 2019 ▸). Several structural techniques have been implemented to break this subatomic resolution barrier and thereby inform protein chemistry, including macromolecular crystallography and, more recently, cryo-electron microscopy (cryo-EM) (Förster & Schulze-Briese, 2019 ▸; Callaway, 2020 ▸). While X-ray crystallography can provide ultrahigh-resolution structural data with the advancement of high-brilliance beams at synchrotron sources, very few H atoms are visible and geometric structure analysis as well as novel computational techniques such as quantum crystallography have to be implemented (Neumann & Tittmann, 2014 ▸; Takaba *et al.*, 2019 ▸; Cachau *et al.*, 2019 ▸). Micro-electron diffraction (microED), which uses crystals smaller than 5 µm, shows promise for H-atom visualization since H atoms scatter electrons more strongly when compared with X-rays (Nannenga & Gonen, 2014 ▸, 2016 ▸). MicroED structures with atomic resolution have been obtained (Luo *et al.*, 2018 ▸; Nannenga, 2020 ▸); however, clear H-atom visualization has thus far been limited to small-molecule structures (Palatinus *et al.*, 2017 ▸). Cryo-EM, a structural technique that does not require a crystalline sample, has made stunning advances in H-atom visualization, with structures of apoferritin and β3 GABA_A_ clearly indicating H-atom positions in omit maps (Yip *et al.*, 2020 ▸; Nakane *et al.*, 2020 ▸). X-ray crystallo­graphy, microED and cryo-EM all suffer from beam-induced radiation damage (Garman & Weik, 2017 ▸; Hattne *et al.*, 2018 ▸; Clabbers & Xu, 2021 ▸). The photosensitive cofactors and metal centres in metalloproteins are particularly sensitive to photo-induced damage (Macedo *et al.*, 2009 ▸; Frankaer *et al.*, 2014 ▸). Photoreduction is observed in multiple structures determined using X-ray radiation, and introduces artefacts that complicate structural and mechanistic interpretation (Bowman *et al.*, 2016 ▸; Corbett *et al.*, 2007 ▸; Wherland & Pecht, 2018 ▸; De la Mora *et al.*, 2012 ▸; Pfanzagl *et al.*, 2020 ▸). Indeed, metal-centre photo-induced damage can be attributed not only to high-energy radiation sources: it has been found that UV–Vis radiation may also induce photoinactivation (Mahor *et al.*, 2020 ▸). Photo-induced damage is often accompanied by changes in bond lengths to the metal centre, a loss/gain in ligands or radiolysis (Horrell *et al.*, 2016 ▸; Yano *et al.*, 2005 ▸; George *et al.*, 2012 ▸; Nass *et al.*, 2015 ▸). X-ray-induced damage can be circumvented by the use of serial femtosecond crystallography (SFX) or serial femtosecond rotation crystallo­graphy (SF-ROX) with an X-ray free-electron laser (XFEL) that provides a brief, intense X-ray pulse to take multiple partial diffraction ‘snapshots’ from many small crystals or one large crystal before significant beam-induced damage (Schlichting, 2015 ▸; Spence, 2017 ▸). A similar serial data-collection approach can be applied to mitigate damage in electron microscopy and diffraction techniques (Nannenga, 2020 ▸; Mastronarde, 2005 ▸; de la Cruz *et al.*, 2019 ▸).

### 1.3. Neutron protein crystallography   

**Figure 1:**
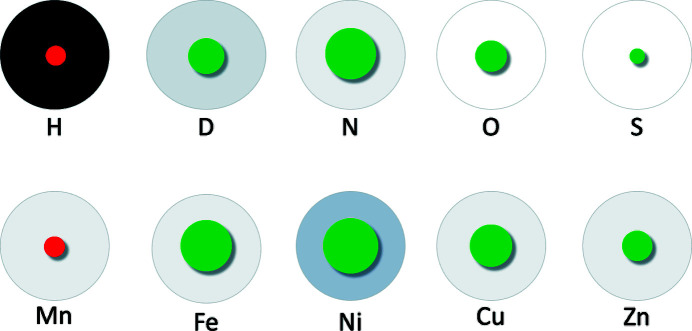
Incoherent neutron scattering cross sections and coherent neutron scattering lengths for selected elements. Relative incoherent scattering cross sections are represented by a disc coloured on a grey scale (dark, high incoherent cross section; light, low incoherent cross section), and relative coherent scattering lengths are represented by red and green discs. The red discs for hydrogen and manganese indicate the negative sign of their scattering lengths, while those shown in green are positive.

In contrast to X-ray diffraction, where the scattering intensity is proportional to the number of electrons, making light elements such as hydrogen poorly visible, the coherent scattering lengths of hydrogen and its isotope deuterium are comparable in magnitude to those of the backbone C, N and O atoms as well as metal atoms such as iron and copper (Fig. 1 ▸; Sears, 1992 ▸; Blakeley *et al.*, 2008 ▸). As a consequence, H/D atoms are visible in neutron scattering-length density (NSLD) maps at moderate resolutions of up to 2.5 Å, making the assignment of protonation states and water-molecule orientations possible (Oksanen *et al.*, 2017 ▸; Schröder *et al.*, 2018 ▸).

In order to successfully perform a neutron protein diffraction experiment, there are technique-inherent limitations that must be taken into consideration. Firstly, since hydrogen has a negative coherent neutron scattering length and a high incoherent cross section, it is necessary to exchange the H atoms present in the protein crystal with the isotope deuterium (D/^2^H; O’Dell *et al.*, 2016 ▸; Meilleur *et al.*, 2009 ▸). This can be accomplished by the exchange of H for D at titratable sites, while non-exchangeable sites remain hydrogenated. H/D exchange can be performed by vapour exchange or soaking of the hydrogenated protein crystal with deuterated crystallization buffer or by directly crystallizing the hydrogenated protein in deuterated buffer (Niimura & Bau, 2008 ▸; Bennett *et al.*, 2008 ▸). An alternative to performing H/D exchange is perdeuteration, in which the non-exchangeable, carbon-bound, H atoms are also exchanged to D by protein expression in deuterated media, which provides a fully deuterated protein sample (Hazemann *et al.*, 2005 ▸). Substitution of H with D results in a decrease in the incoherent scattering cross section from 80.26 to 2.05 barns, which results in an improved signal and a reduction of background noise during data collection (Fig. 1 ▸). Furthermore, the negative coherent neutron scattering length of H, with a value of −3.74 fm, in contrast to the positive scattering lengths of D, C, N and O (Fig. 1 ▸) leads to density cancellation in NSLD maps which can complicate interpretation. While H/D exchange partially alleviates NSLD map cancellations, cancellation can be fully circumvented by the use of perdeuterated protein samples, which result in continuous NSLD maps, thereby aiding the modelling of side chains. However, it should be noted that protein perdeuteration results in lower yields and may impede crystallization.

Secondly, in metalloproteins, metals with weak positive or negative scattering lengths, such as zinc (5.68 fm) or manganese (−3.73 fm), are poorly visible in NSLD maps. It is therefore advantageous to perform a joint refinement in which the X-ray data can be used to determine the metal-atom position and identity. X-ray diffraction data are typically collected following neutron data collection using the same crystal or using a different crystal grown under identical conditions. The two data sets are subsequently used in a joint refinement in which the coordinates of the heavy atoms of the protein backbone and side chain are refined against the X-ray data, while the neutron data are used to determine the H/D positions (Adams *et al.*, 2009 ▸). X-ray data sets collected from metalloproteins should however be collected using the lowest X-ray dose possible to limit radiation-induced damage. It is additionally advisable to perform an X-ray radiation dose-exposure series to establish specific effects that X-ray beam exposure may have on the metallocenter. This will assist in the identification of any X-ray-induced artefacts during joint refinement. Thirdly, since the flux of neutron sources is low when compared with high-brilliance X-ray sources or even home-source X-ray generators, large protein crystals with a minimum volume of 0.1 mm^3^ are required and data collection requires several days (Ng *et al.*, 2015 ▸; Blakeley *et al.*, 2004 ▸). The interested reader is referred to the extensive descriptions of the technique by O’Dell *et al.* (2016 ▸) and Schröder & Meilleur (2020 ▸) for practical applications.

## 2. Neutron protein crystallography of transition-state metal oxidoreductases   

The sensitivity of neutron protein crystallography to the positions of H atoms, while not inducing radiation damage, makes it an ideal technique to probe the structure of metalloproteins. Presented here is an overview of the structural and mechanistic insights gained from neutron protein diffraction of various transition-metal-containing proteins in the oxido­reductase (EC 1) family (Table 1 ▸). While these examples demonstrate the power of neutron protein diffraction to characterize the active site and reaction intermediates, they also illustrate the value of neutron protein crystallography as a complementary tool. Neutron protein crystallography provides an all-atom model of the metalloprotein active site and intermediates which can inform quantum-chemistry calculations of the electronic rearrangements and bond breakage and formation in the metalloprotein redox mechanism. Metalloproteins can be modelled using density-functional theory (DFT) calculations to determine the energetics of a limited number of atoms, often confined to active-site residues and intermediates (Siegbahn & Himo, 2009 ▸; Siegbahn, 2021 ▸). Quantum-mechanics/molecular-mechanics (QM/MM) modelling of metalloproteins uses QM to model the electronic structure of the active site and MM, described by a force field, to model the effects of the surrounding protein environment (van der Kamp & Mulholland, 2013 ▸). Both DFT and QM/MM calculations are computationally expensive, making complete knowledge of the active site, particularly protonation states and hydrogen-bond orientations, important to inform the calculations and minimize the number of input models. The use of additional techniques such as XFELs, spectroscopy, mutagenesis and kinetic studies provide a complete picture of the underlying enzyme chemistry, as will be discussed herein.

### 2.1. Copper nitrite reductase   

**Figure 2:**
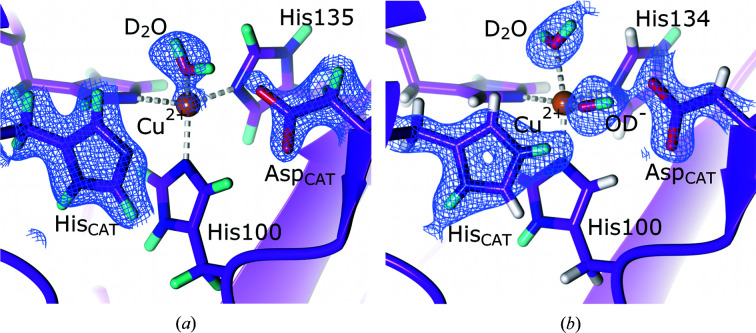
The active site of copper nitrite reductase (PDB entry 6gtj, perdeuterated; Halsted *et al.*, 2019 ▸). (*a*) A D_2_O molecule is bound to the active site with a neutral His256 (His_CAT_). 2*F*_o_ − *F*_c_ NSLD map (σ = 1.00) is displayed as a blue mesh; H and D atoms are displayed in white and turquoise, respectively. (*b*) An OD^−^ ion is bound to the active site with a positively charged His211 (His_CAT_; PDB entry 6l46, H/D exchanged; Fukuda *et al.*, 2020 ▸). NSLD 2*F*_o_ − *F*_c_ density (σ = 1.50) is displayed as a blue mesh; H and D atoms are displayed in white and turquoise, respectively.

Copper nitrite reductases (CuNiRs; EC 1.7.2.1) form part of the anaerobic respiratory pathway of denitrification (Zumft, 1997 ▸). During denitrification, microbes use nitrate (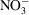) as an electron acceptor in a four-step reductase-dependent process composed of the sequential formation of 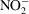, NO and N_2_O and the final release of N_2_ (Yang *et al.*, 2020 ▸). CuNiRs are periplasmic enzymes that catalyze the reduction of 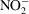 by copper(I) with the subsequent addition of two protons to form water and NO as follows (Eady & Hasnain, 2003 ▸):
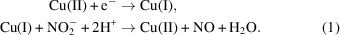



The structure of CuNiRs has been well characterized, consisting of a homotrimer which contains two cupredoxin-type domains: a type I (T1Cu) domain involved in electron transfer from an electron-donor partner protein to a type II (T2Cu) catalytic domain (Horrell *et al.*, 2017 ▸; Merkle & Lehnert, 2012 ▸). The copper of the T1Cu domain is coordinated by two histidine residues, a cysteine residue and a methionine residue in a tetrahedral geometry (Godden *et al.*, 1991 ▸). The catalytic T2Cu centre is located ∼12 Å from T1Cu and the two sites are connected via an electron-transfer bridge composed of cysteine and histidine. The T2Cu copper is coordinated by three histidine residues in the equatorial plane. Of particular catalytic importance are the active-site residues Asp_CAT_ and His_CAT_, which play a role in proton transfer and substrate binding and are connected by a bridging water molecule via hydrogen bonds (Kataoka *et al.*, 2000 ▸; Boulanger *et al.*, 2000 ▸). Extensive structural studies have been performed on CuNiRs to determine their catalytic mechanism; however, X-ray-induced reduction results in structural changes and unintended redox reactions (Murphy *et al.*, 1997 ▸; Leferink *et al.*, 2011 ▸; Tocheva *et al.*, 2004 ▸, 2007 ▸; Antonyuk *et al.*, 2005 ▸; Nojiri *et al.*, 2009 ▸; Hough *et al.*, 2008 ▸). To circumvent the X-ray-induced radiation damage, structures were obtained using XFEL crystallography (Fukuda, Tse, Suzuki *et al.*, 2016 ▸; Fukuda, Tse, Nakane *et al.*, 2016 ▸; Horrell *et al.*, 2017 ▸; Halsted *et al.*, 2018 ▸). To further characterize the mechanism of CuNiR by investigating active-site protonation states and substrate binding in the absence of radiation damage, Halsted and coworkers collected serial femtosecond rotational crystallo­graphy (SF-ROX) data for oxidized, reduced and substrate-bound forms of *Achromobacter cycloclastes* CuNiR (*Ac*NiR) as well as a room-temperature neutron protein diffraction structure of the oxidized form (Halsted *et al.*, 2019 ▸). SF-ROX is an approach that utilizes XFEL data collection by performing consecutive exposures following stepwise rotation and translation along a large crystal with known orientation, in contrast to the single-use exposure of randomly oriented microcrystals (Schlichting, 2015 ▸). Asp_CAT_ is known to be present in two conformations in CuNiRs: a ‘proximal’ orientation facing T2Cu and hydrogen-bonded to His_CAT_ via the bridging water, and a ‘gatekeeper’ orientation facing away from T2Cu and hydrogen-bonding to the copper water ligand (Antonyuk *et al.*, 2005 ▸). Analysis of the SF-ROX data for the oxidized form revealed a T2Cu active site with Asp_CAT_ in the proximal conformation with two orientations, as opposed to the single proximal orientation observed from the synchrotron data. In contrast, the neutron data collected at 1.9 Å resolution indicated a proximal Asp_CAT_ with only one orientation. Unexpectedly, several previously determined XFEL structures have noted the absence of the T2Cu axial water ligand (Fukuda, Tse, Suzuki *et al.*, 2016 ▸; Fukuda, Tse, Nakane *et al.*, 2016 ▸; Halsted *et al.*, 2018 ▸); however, the SF-ROX data presented by Halsted and coworkers indicated that a water molecule is coordinated to T2Cu in the axial position, which is confirmed by analysis of the NSLD maps (Fig. 2 ▸ *a*). The NSLD maps further indicate that Asp_CAT_ is deprotonated, while His_CAT_ is protonated on N^δ2^ at the catalytically optimal pD of 5.4, in contrast to previous spectroscopic and computational studies, which proposed that the resting-state His_CAT_ is doubly protonated (Ghosh *et al.*, 2009 ▸). The NSLD maps additionally support this single protonation state of His_CAT_ by confirming the positioning of the bridging water as a hydrogen-bond donor to both Asp_CAT_ and N^ɛ1^ of His_CAT_. The authors conclude that the observed protonation states in the *Ac*NiR active site support the hypothesis that upon binding of the nitrite substrate and displacement of the axial water ligand, protonation of Asp_CAT_ is triggered via the bridging water. This protonation of Asp_CAT_ initiates proton-coupled electron transfer from T1Cu and subsequent catalysis (Brenner *et al.*, 2009 ▸; Ghosh *et al.*, 2009 ▸)

An additional CuNiR neutron protein crystallography structure was subsequently solved from *Geobacillus thermodenitrificans* (*Gt*NiR) at 1.5 Å resolution (Fukuda *et al.*, 2020 ▸). Contrary to the findings on *Ac*NiR by Halsted and coworkers, two ligands were observed coordinated to T2Cu. One of these ligands is an equatorial water, while the second was fitted as a two-atom axial hydroxide ligand according to the NSLD maps (Fig. 2 ▸ *b*). The Asp_CAT_ residue was in the ‘proximal’ conformation in one orientation only and was found to be deprotonated, in agreement with previous findings; however, the His_CAT_ residue was found to be doubly protonated at the measured pD of 5.3. In this *Gt*NiR structure, at an improved resolution, the bridging water acts as a hydrogen-bond donor to Asp_CAT_, while accepting a hydrogen bond from N^ɛ1^ of His_CAT_. The authors reason that the difference in protonation may be due to a proton exchange between His_CAT_ and the axial water observed in *Ac*NiR. The presence of a stable hydroxide ligand in the neutron structure is similar to the hydroxide ligand observed bound to T2Cu in computational studies, suggesting the direct release of NO without forming a copper–nitrosyl intermediate (Lintuluoto & Lintuluoto, 2016 ▸; Ghosh *et al.*, 2009 ▸; Qin *et al.*, 2017 ▸). The authors therefore conclude that their structure shows that nitrite reduction proceeds without the formation of a copper–nitrosyl intermediate, although this is in disagreement with NO-soaked and multiple serial structures from single-crystal (MSOX) studies that indicate a copper–nitrosyl species (Tocheva *et al.*, 2004 ▸, 2007 ▸; Merkle & Lehnert, 2009 ▸; Horrell *et al.*, 2018 ▸). The level of H/D exchange was further analyzed to draw conclusions on the dynamics of the structure, particularly as it pertains to the electron-transfer pathway from T1Cu to T2Cu. The bridging histidine that links the two copper sites shows comparatively low H/D exchange at its N^δ2^ site (low D-atom occupancy), which suggests that electron transfer proceeds across this rigid hydrogen bond linking the cysteine backbone carbonyl and the histidine N^δ2^ hydrogen. Such an electron jump across a rigid hydrogen bond has been proposed by DFT calculations, and these neutron diffraction structural findings represent the first experimental evidence thereof (Hadt *et al.*, 2014 ▸).

Neutron protein diffraction studies provide valuable insight into protein chemistry; however, it is also useful to use further complementary techniques to obtain a more complete understanding of the system. Such is the case for CuNiR, in which recently solved atomic resolution damage-free XFEL structures revealed new mechanistic insights which provide a new perspective on the neutron diffraction findings (Rose *et al.*, 2021 ▸). Rose and coworkers used advanced synchrotron-radiation and XFEL data to obtain substrate-free, substrate-bound and product-bound structures of a *Bradyrhizobium* copper nitrite reductase (*Br* ^2D^NiR). Using unrestrained refinement of their high-resolution structures, they show that His_CAT_ is singly protonated on N^δ2^, in agreement with Halsted *et al.* (2019 ▸). Rose and coworkers also find that Asp_CAT_ is in the ‘proximal’ conformation with two orientations, as seen in *Ac*NiR. To generate the *Br* ^2D^NiR enzyme–product complex, crystals were soaked with nitrite and reduced with dithionite, after which XFEL data were collected. Analysis revealed the presence of a copper–nitrosyl species, refuting the hypothesis that NO is immediately released following proton-coupled electron transfer, leaving a hydroxide-bound copper intermediate (Fukuda *et al.*, 2020 ▸). The presence of the hydroxide intermediate in *Gt*NiR is suggested to be an artefact which can be attributed to the additional artificial coppers that are present in the neutron structure. These damage-free XFEL and neutron diffraction data findings highlight the strengths of multiple techniques to gain insight into the CuNiR reaction mechanism while also allowing previous experimental findings to be critically evaluated.

### 2.2. Copper amine oxidase   

Copper amine oxidases (CAOs; EC 1.4.3.6) are a class of redox enzymes that are present in prokaryotes and eukaryotes and are involved in the oxidative deamination of primary amines (Dooley *et al.*, 1993 ▸). The overall reaction of CAOs results in the formation of an aldehyde from its corresponding primary amine, after which molecular oxygen is reduced to hydrogen peroxide and ammonia is released to restore the enzyme resting state according to the general equation given below (Wilmot *et al.*, 1997 ▸):
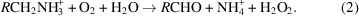



CAOs were initially described as ‘pink enzymes’ due to the 2,4,5-trihydroxyphenylalanine quinone (TPQ) cofactor derived from a post-translationally modified tyrosine present in the active site with a visible absorbance peak at 480 nm (Floris & Mondovi, 2009 ▸). The structure of CAOs has been characterized as a mushroom-shaped homodimer with a molecular mass of 70–95 kDa, with the active site located in a C-terminal β-sandwich domain (Parsons *et al.*, 1995 ▸). CAO catalysis is classified as a ping-pong bi-bi mechanism composed of an oxidative and a reductive half-reaction (Bardsley *et al.*, 1974 ▸; Brazeau *et al.*, 2004 ▸). In the reductive half-reaction, the resting-state TPQ cofactor present in its oxidative enolate form, TPQ_ox_, is nucleophilically attacked by the substrate amine (Medda *et al.*, 1995 ▸). Following formation of the substrate and product Schiff bases (TPQ_ssb_ and TPQ_psb_, respectively) the aldehyde product is released, resulting in the formation of aminoresorcinol (TPQ_amr_) in equilibrium with the semiquinone radical (TPQ_sq_) (Klema & Wilmot, 2012 ▸):
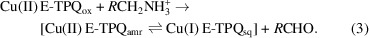



The oxidative half-reaction proceeds by oxidation of TPQ_sq_ to the iminoquinone (TPQ_imq_) by two-electron reduction and protonation of molecular oxygen to form hydrogen peroxide:
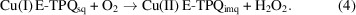



Hydrolysis of TPQ_imq_ results in formation of the resting-state TPQ_ox_ with the concomitant release of ammonia:
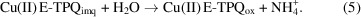



The active-site copper is believed to play no role in the reductive half-reaction (Rinaldi *et al.*, 1984 ▸). During the oxidative half-reaction, the copper is involved in mediating electron transfer between TPQ_amr_ and dioxygen and binding the reduced dioxygen species (Kishishita *et al.*, 2003 ▸). The CAO reaction mechanism involves several intermediates in which proton transfer and protonation states play an important role (Chiu *et al.*, 2006 ▸; Murakawa *et al.*, 2015 ▸).

**Figure 3:**
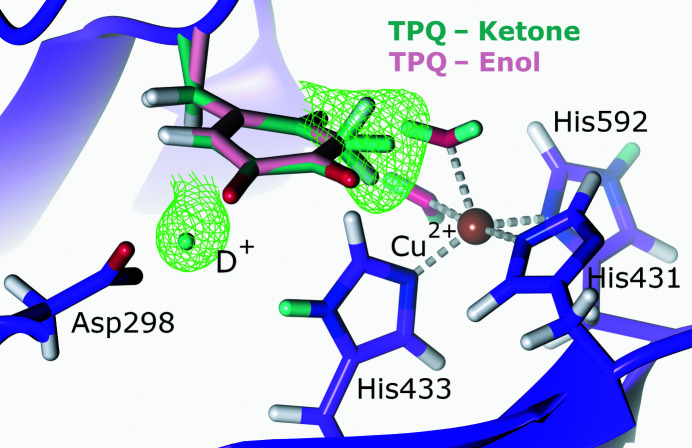
The active site of copper amine oxidase. The cofactor TPQ is present in its ketone and enol forms and a proton is shared between TPQ and the active-site Asp298 (PDB entry 6l9c, H/D exchanged; Murakawa *et al.*, 2020 ▸). *F*_o_ − *F*_c_ NSLD omit map (σ = 3.00) is displayed as a green mesh for selected D atoms; H and D atoms are displayed in white and turquoise, respectively.

To further characterize the active site of CAO from *Arthrobacter globiformis* (AGAO), Murakawa and coworkers collected a 1.72 Å resolution neutron data set of resting-state AGAO together with a 1.14 Å resolution X-ray diffraction data set for joint refinement (Murakawa *et al.*, 2020 ▸). Analysis of the NSLD maps revealed that the resting-state enolate (TPQ_ox_) was in equilibrium with its keto form (Fig. 3 ▸). This was evident from the ellipsoidal positive NSLD difference density peak at the C3 position of TPQ, indicating mixed CD and CD_2_ states characteristic of the enolate and ketone forms, respectively. The high-resolution X-ray diffraction data indicated that the TPQ quinone ring had a bent conformation, which was further supported by DFT calculations, which indicated 59% keto and 41% enolate occupancy. Aspartic acid has been proposed to function as a catalytic base by abstracting a proton from the substrate amine and the substrate Schiff base (TPQ_ssb_), as well as during the hydrolysis of TPQ_imq_ to TPQ_ox_ (Murray *et al.*, 1999 ▸; Chiu *et al.*, 2006 ▸). In their neutron diffraction structure, Murakawa and coworkers emphasize the capability of neutron diffraction to reveal new and exciting conformations by presenting evidence for a triply shared proton between TPQ and the conserved aspartic acid residue Asp298 in a trifurcated hydrogen bond identified by a positive NSLD difference density peak. Although an uncommon protonation state, a triply shared proton is not unprecedented, having been observed in a 5′-methylthio­adenosine nucleosidase (Banco *et al.*, 2016 ▸). However, a subsequent QM/MM study investigating the observed protonation states of Asp298 and TPQ found that a triply shared proton was energetically unfavourable, with bonding to one of the two carboxyl O atoms representing a more stable conformation (Shoji *et al.*, 2020 ▸). Although computational studies found this triply shared proton to be unstable, neutron diffraction data corroborate the role of Asp298 as a catalytic base with a highly reactive proton.

The AGAO active-site copper is coordinated by two water molecules (W_ax_ and W_eq_) and three histidine residues (His431, His433 and His592) with square-pyramidal geometry (Wilce *et al.*, 1997 ▸). Murakawa and coworkers assigned His431 as a negatively charged imidazolate anion, potentially due to the ability of positively charged metal ions to significantly lower the apparent p*K*_a_ of imidazole rings, thereby resulting in deprotonation (Hasegawa *et al.*, 2000 ▸). Similarly, the coordinating W_ax_ was modelled in its deprotonated state as a hydroxide anion, which potentially donates a proton to the enolate form of TPQ to form the observed mixed-state keto form. Additional QM/MM calculations suggest that His431 is potentially present as a imidazolate ion dependent on the surrounding hydrogen-bonding network, demonstrating the importance of these networks and protonation pathways in the reaction mechanism (Shoji *et al.*, 2020 ▸). The QM/MM calculations could not definitively show a stabilized W_ax_ deprotonated as a hydroxide anion. Despite requiring further experimental and computational data to fully elucidate the reaction mechanism of AGAO and the unique protonation states observed in the AGAO neutron diffraction structure, these results provide an informative perspective on copper-containing amine oxidases and will help to inform on enzymes displaying similar chemistry.

### 2.3. Heme peroxidases   

**Figure 4:**
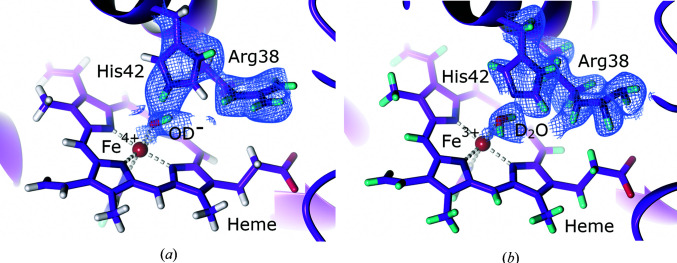
The active site of the heme centre of ascorbate peroxidase (APX). (*a*) Compound II of APX with a positively charged His42 (PDB entry 5jpr, H/D exchanged; Kwon *et al.*, 2016 ▸). 2*F*_o_ − *F*_c_ NSLD map (σ = 1.50) is displayed as a blue mesh; H and D atoms are displayed in white and turquoise, respectively. (*b*) A neutral Arg38 residue in ascorbate-bound APX (PDB entry 6xv4, perdeuterated; Kwon *et al.*, 2020 ▸). 2*F*_o_ − *F*_c_ NSLD map (σ = 1.00) is displayed as a blue mesh; H and D atoms are displayed in white and turquoise, respectively.

Heme peroxidases catalyze the hydrogen peroxide-dependent oxidation of a variety of substrates (Poulos, 2010 ▸). Oxygen activation during catalysis involves the formation of two oxidized ferryl [iron(IV)] heme intermediates termed Compound I and Compound II (Keilin & Hartree, 1951 ▸; George, 1952 ▸):
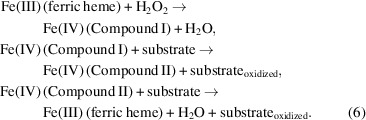



Compound I forms first in the reaction cycle and contains either a porphyrin π-cation radical or a protein radical, while further one-electron reduction results in the formation of Compound II (Dawson, 1988 ▸). The chemical nature of the ferryl species has been an area of extensive research, with debate on whether the species should be characterized as a deprotonated iron(IV)-oxo species [Fe(IV)=O] or a protonated iron(IV) hydroxide [Fe(IV)—OH] (Groves & Boaz, 2014 ▸; Groves, 2014 ▸; Sligar, 2010 ▸; Karlin, 2010 ▸). The identity of the ferryl intermediate has been probed using multiple spectroscopic methods, including resonance Raman, EXAFS and Mössbauer; however, none were definitive (Behan & Green, 2006 ▸; Terner *et al.*, 2006 ▸). Structural studies using X-ray crystallography sought to determine the identity by differentiating between the shorter Fe(IV)=O and longer Fe(IV)—OH Fe–O atom distances; however, in-beam photoreduction results in an increased bond length, which complicates species assignment (Hersleth *et al.*, 2006 ▸; Meharenna *et al.*, 2010 ▸). An important step towards unravelling the identity of the ferryl species came with neutron protein diffraction studies on the class I heme peroxidases cytochrome *c* peroxidase (C*c*P; EC 1.11.1.5) and ascorbate peroxidase (APX; EC 1.11.1.11) (Moody & Raven, 2018 ▸). C*c*P and APX are intracellular proteins that contain a heme iron coordinated to a proximal histidine residue (Poulos *et al.*, 1980 ▸; Jones *et al.*, 1998 ▸). C*c*P is present in the mitochondria and plays a role in the electron-transport chain by catalyzing the reduction of H_2_O_2_ to water by receiving reducing equivalents from cytochrome *c* (C*c*; Volkov *et al.*, 2011 ▸). Upon the reaction of ferric C*c*P with H_2_O_2_, Compound I is formed with the radical located on a tryptophan residue adjacent to the heme (Sivaraja *et al.*, 1989 ▸). Casadei and coworkers successfully used neutron protein diffraction to characterize the ferric form of C*c*P at 2.4 Å resolution at room temperature and were able to identify the protonation state of the ferryl Compound I species under cryoconditions with a structure at 2.5 Å resolution (Casadei *et al.*, 2014 ▸). Analysis of the NSLD maps revealed that the ferric form contained a water coordinated to the heme on the distal face as well as a neutral distal histidine. Joint refinement of cryo-trapped Compound I established the identity of the ferryl species as the deprotonated Fe(IV)=O and indicated a positively charged, doubly protonated distal histidine: a thus far unprecedented finding since it had been assumed that both protons of H_2_O_2_ were used in formation of the water molecule. Although the iron–oxygen bond length could not be accurately determined at the resolution of the neutron structures, NSLD omit maps clearly indicate a deprotonated Compound I species. This calls into question the previously established role of the distal histidine as a peroxide-deprotonating base catalyst and subsequent acid catalyst for water formation to form Compound I (Vidossich *et al.*, 2010 ▸). Casadei and coworkers posit that an additional proton is needed for Compound I formation, similar to Compound I formation in cytochrome P450s (Shaik *et al.*, 2005 ▸). Following the identification of Compound I, Kwon and coworkers were able to trap Compound II of APX in a further cryo-neutron diffraction study (Kwon *et al.*, 2016 ▸). APX catalyzes the H_2_O_2_-dependent oxidation of ascorbate, resulting in the formation of water and monodehydroascorbate (Asada, 1992 ▸). The formation of Compound I is rapid; however, the subsequent decay into Compound II is stable over a sufficient timespan to allow cryo-trapping. Analysis of the 2.2 Å resolution APX Compound II NSLD maps is consistent with the presence of a protonated Fe(IV)—OH species (Fig. 4 ▸ *a*). The distal histidine residue was also found to be doubly protonated and positively charged. The presence of an Fe(IV)—OH species, although supported by convincing NSLD maps, is suggested to be unlikely because an electron-donating proximal ligand such as the thiolate in cytochrome P450s is necessary as opposed to the histidine proximal ligand in heme peroxidases (Yosca *et al.*, 2013 ▸). This is supported by a recent study that utilized Mössbauer and X-ray absorption spectroscopy to show that the APX Compound II species is an unprotonated Fe(IV)=O species (Ledray *et al.*, 2020 ▸).

A matter of contention is the 1.88 Å iron–oxygen bond length observed in the neutron study, which is considered to be much longer than the 1.76 Å bond length expected for a histidine-coordinated iron(IV) species and suggests an iron(III) hydroxide species (Stone *et al.*, 2006 ▸; Green, 2006 ▸). The strengths of neutron diffraction in providing insight into the presence of light atoms and allowing conclusions on protonation states can be juxtaposed with its weakness in assigning metal oxidation states, making the use of complementary techniques such as spectroscopy crucial. A closer examination of the bond lengths in Compound II of C*c*P and APX was performed by Kwon and coworkers using radiation-damage-free XFEL diffraction to accurately establish the iron–oxygen bond length (Kwon *et al.*, 2021 ▸). C*c*P displays an iron–oxygen distance of 1.76 Å, while APX has a bond length of 1.87 Å. The range of bond lengths observed led Kwon and coworkers to conclude that the bond length may ‘flex’ due to protein dynamic motions in the heme active site. Indeed, a distal arginine residue in APX has been suggested to be dynamic and to be involved in proton delivery to the active-site heme (Efimov *et al.*, 2011 ▸), and the XFEL study indicates that the arginine has both heme-facing ‘in’ and heme-distant ‘out’ conformations. A 2.1 Å resolution neutron protein diffraction structure of ferric APX bound to ascorbate obtained by Kwon and coworkers indicates that this distal arginine is neutral, supporting its role as a dynamic proton donor (Fig. 4 ▸ *b*; Kwon *et al.*, 2020 ▸). The dynamic hydrogen-bonding rearrangement observed in APX is not observed in C*c*P, where the distal arginine is present in only one conformation. This may explain the longer iron–oxygen bond distance in APX when compared with C*c*P, since the more dynamic heme hydrogen-bonding environment may fine-tune the ferryl species. The implications that such fine-tuning may have for heme reactivity in different peroxidases remains to be established; however, these findings highlight the effect that the active-site environment can have on protein reactivity and selectivity.

### 2.4. Lytic polysaccharide monooxygenase   

The ability of enzymes to efficiently catalyze complex reactions under mild reaction conditions when contrasted with traditional chemical processes has made them invaluable for the production of value-added compounds (Wiltschi *et al.*, 2020 ▸). In particular, the efficient degradation of cellulose by biocatalytic means for bioethanol production is increasingly coming to the forefront as demands for sustainable energy production rise (Liao *et al.*, 2016 ▸). Fungi, in particular, employ numerous glycoside hydrolases to degrade lignocellulose as an energy source (Lynd *et al.*, 2002 ▸). These glycoside hydrolases have been employed in commercial cellulose saccharification; however, the complex structure of lignocellulose, particularly its biotic attack-resistant crystalline cellulose core, has limited solubilized sugar yields (Harris *et al.*, 2014 ▸; Cragg *et al.*, 2015 ▸). However, a class of copper-dependent oxidoreductases termed lytic polysaccharide monooxygenases (LPMOs; EC 1.14.99.56) were discovered that resulted in increased cellulose yields by disrupting the crystalline cellulose structure by oxidative cleavage (Himmel *et al.*, 2007 ▸; Bissaro *et al.*, 2018 ▸). Fungal LPMOs are secreted proteins with a planar active site located on the enzyme surface for carbohydrate substrate binding (Tandrup *et al.*, 2018 ▸). The active-site copper is coordinated by a characteristic ‘histidine brace’ in the equatorial plane composed of the N-terminal histidine amino group and imidazole-ring N atom and a further imidazole N atom from a second conserved histidine (Quinlan *et al.*, 2011 ▸). LPMOs perform oxidative glycosidic bond cleavage by catalyzing hydroxylation of the C1 or C4 position of the glycosidic bond (Frommhagen *et al.*, 2018 ▸). In the O_2_-dependent pathway, the reaction is initiated by one-electron reduction of the resting-state copper(II) to copper(I), after which oxygen binds and is spontaneously reduced to superoxide (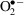). Rapid superoxide formation following one-electron reduction of an equatorially bound oxygen by copper(I) has been shown by electron paramagnetic resonance (EPR) and stopped-flow absorption spectroscopy studies, and was supported by DFT studies with coordination geometry informed by X-ray absorption near-edge structure (XANES) and extended X-ray absorption fine structure (EXAFS) of the oxidized and reduced copper centre (Kjaergaard *et al.*, 2014 ▸). Following superoxide formation, the LPMO requires further precisely timed reduction and protonation steps to form the hydroxylated product, as illustrated in the overall reaction below; however, the intermediates involved in the mechanism remain an area of active experimental and computational investigation (Walton & Davies, 2016 ▸).
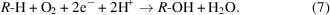



**Figure 5:**
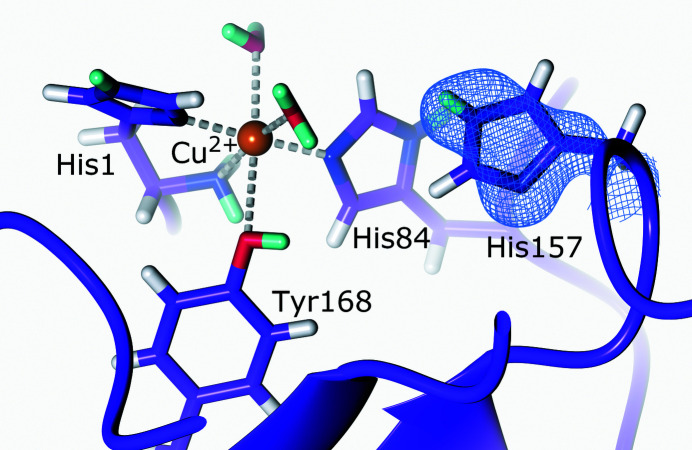
The active site of LPMO. The second-shell His157 is neutral in the copper(II) resting state (PDB entry 5tki, H/D exchanged; O’Dell *et al.*, 2017 ▸). 2*F*_o_ − *F*_c_ NSLD map (σ = 1.50) is displayed as a blue mesh; H and D atoms are displayed in white and turquoise, respectively.

An essential step in elucidating the LPMO mechanism is the characterization of the H atom-abstracting species (HAA), a highly reactive species that abstracts an H atom from the glycosidic carbon to be hydroxylated (Meier *et al.*, 2018 ▸). Multiple candidates have been proposed using structural, spectroscopic, small-molecule copper complex and computational studies, including superoxide (Phillips *et al.*, 2011 ▸; Beeson *et al.*, 2012 ▸; Li *et al.*, 2012 ▸), hydroperoxy (Neisen *et al.*, 2017 ▸), hydroxy (Dhar & Tolman, 2015 ▸) and oxyl species (Walton & Davies, 2016 ▸; Kim *et al.*, 2014 ▸; Lee & Karlin, 2015 ▸; Wang *et al.*, 2018 ▸, 2020 ▸). Additionally, it has been proposed that hydrogen peroxide (H_2_O_2_) acts as the co-substrate in a reaction mechanism that proceeds via an oxyl HAA intermediate (Bissaro *et al.*, 2017 ▸; Hangasky *et al.*, 2018 ▸). This reaction mechanism requires the resting-state copper(II) to be reduced to copper(I) in a ‘priming reduction’, after which multiple hydroxylation reactions can be catalyzed:
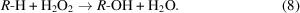



In an initial study to investigate oxygen binding in LPMOs, Bacik and coworkers collected a 1.1 Å resolution X-ray diffraction data set and a 2.1 Å resolution neutron diffraction data set from a chitin-binding LPMO from *Jonesia denitrificans* (*Jd*LPMO10A; Bacik *et al.*, 2015 ▸, 2017 ▸). The X-ray structure was interpreted as containing an oxygen cofactor modelled as a peroxide species coordinated in the equatorial plane. The crystal asymmetric unit contained two molecules, molecules *A* and *B*, related by noncrystallographic symmetry. The dioxygen species was modelled with a side-on coordination to the molecule *A* copper and an end-on coordination to the molecule *B* copper. In the separately solved neutron structure, only molecule *B* was modelled with an end-on coordinated dioxygen species; however, the limited resolution did not permit an unambiguous determination of the chemical nature of the dioxygen species. The X-ray and neutron data deposited by Bacik and coworkers were later revisited by Caldararu and coworkers, who performed a joint X-ray–neutron quantum refinement of the originally deposited data (Caldararu *et al.*, 2019 ▸). The joint quantum refinement noted a discrepancy in the positioning of the dioxygen species, with the molecule *A* dioxygen more resembling an end-on positioning following the introduction of a QM potential. Subsequent vacuum QM and QM/MM calculations informed by the quantum-refined X-ray data-only structure suggest that the dioxygen species may be a superoxo species; however, the discrepancy of the binding modes of the dioxygen species in molecules *A* and *B*, the weak nuclear density of the dioxygen species in molecule *A* and the absence of a reducing agent for one-electron reduction of the resting-state copper(II) to ensure dioxygen binding made these initial findings of dioxygen activation ambiguous. To further investigate dioxygen activation at the LPMO active site and identify the activated oxygen species involved in the initial steps of catalysis, O’Dell and coworkers collected both high-resolution X-ray and room-temperature neutron diffraction data sets for LPMO9D from *Neurospora crassa* (*Nc*LPMO9D; O’Dell *et al.*, 2017 ▸). In order to characterize the activated oxygen species, *Nc*LPMO9D crystals were chemically reduced to copper(I) and the resulting intermediate was freeze-trapped prior to X-ray diffraction data collection. Structural analysis of the 1.20 Å resolution data revealed the first evidence for a dioxygen species (potentially superoxo or peroxo) coordinated to the copper with η_1_ end-on geometry: an exciting development in the characterization of the early steps of LPMO dioxygen activation. Analysis of the second *Nc*LPMO9D molecule in the crystal structure related by noncrystallographic symmetry showed that it did not contain a coordinated activated dioxygen species; however, an oxygen could be modelled in a ‘pre-binding’ conformation, presumably prior to coordinating at the equatorial position. To gain more detailed insight into the protonation state of second-shell residues that may play a role in catalysis, a 2.12 Å resolution structure of the copper(II) resting-state form of *Nc*LPMO9D was solved using room-temperature neutron protein crystallography. Analysis of the NSLD maps collected at room temperature revealed that His157 is singly protonated on N^ɛ2^ (Fig. 5 ▸). His157 is a second-shell residue pointing towards the oxygen pre-binding site and may therefore play a role in promoting oxygen activation. The NSLD maps did not indicate the presence of a pre-bound oxygen; however, the data were collected at pH 5.6 (pD 6.0), while LPMOs perform catalysis in a more acidic environment of pH ∼5 where cellulases show optimum activity (Schülein, 1997 ▸; Boer & Koivula, 2003 ▸; Cragg *et al.*, 2015 ▸). It is therefore posited that under such acidic conditions His157 would be positively charged and doubly protonated on N^ɛ2^ and N^δ1^, which may promote oxygen binding. DFT calculations confirmed that while oxygen binding to the neutral His157 is thermoneutral, oxygen binding to the positively charged His157 is highly thermodynamically favourable.

These findings show that His157 may play a role in ensuring efficient catalysis and illustrate the importance of second-shell residues. Indeed, DFT calculations of the LPMO mechanism assign this second-shell histidine a positive charge and have postulated that it may play a role as a proton donor to active-site intermediates (Span *et al.*, 2017 ▸; Hedegård & Ryde, 2018 ▸). The exact mechanism by which LPMOs achieve catalysis remains to be elucidated and the poorly soluble carbohydrate substrate presents challenges in crystallographic structural analysis, although a limited number of structures have been determined (Frandsen *et al.*, 2016 ▸; Simmons *et al.*, 2017 ▸; Tandrup *et al.*, 2020 ▸). Future spectroscopic, computational and structural studies, including neutron protein diffraction studies investigating the *Nc*LPMO9D structure under functionally relevant acidic conditions and bound to a cryo-trapped intermediate (Schröder *et al.*, 2021 ▸), may help to shed light on LPMO catalysis.

### 2.5. Manganese superoxide dismutase   

Reactive oxygen species (ROS) play a role in cellular signalling; however, accumulation of these oxygen ions or radicals can result in cellular and organ damage (Auten & Davis, 2009 ▸). Superoxide dismutases (SODs) are metallo­enzymes that function to mitigate the damage from ROS by converting superoxide (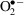) into oxygen (O_2_) or hydrogen peroxide (H_2_O_2_) (Fridavich, 1995 ▸). The active site of SODs can contain an iron, manganese, copper or zinc cofactor dependent on the taxonomic domain (Abreu & Cabelli, 2010 ▸). In eukaryotes, manganese superoxide dismutase (MnSOD; EC 1.15.1.1) is situated in the mitochondrial matrix, where it prevents the accumulation of superoxide formed due to mitochondrial electron leaks (Jastroch *et al.*, 2010 ▸). MnSOD is functional as a homotetramer, with the active-site manganese ion coordinated to His26, His74, Asp159 and His163 (Borgstahl *et al.*, 1992 ▸). The reaction mechanism of MnSOD proceeds by cycling through a manganese(III) and manganese(II) oxidation state as shown below (Holm *et al.*, 1996 ▸):
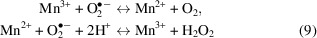



Manganese(III) oxidizes superoxide to molecular oxygen and the formed manganese(II) reduces the superoxide species with concomitant protonation to form hydrogen peroxide (Miller *et al.*, 2003 ▸). The MnSOD mechanism requires concerted proton and electron transfer (CPET) to achieve a thermodynamically favourable reaction during catalysis, as has been observed for multiple enzymes involved in redox reactions (Chang *et al.*, 2004 ▸). In addition to the active-site residues, mutagenesis, structural and computational studies have shown that second-shell residues, including His30, Tyr34, Gln143, Glu162 and Tyr166, play an important role in the active-site metal redox cycling and protonation (Guan *et al.*, 1998 ▸; Srnec *et al.*, 2009 ▸; Heimdal *et al.*, 2011 ▸; Hsieh *et al.*, 1998 ▸; Hearn *et al.*, 2003 ▸; Perry *et al.*, 2009 ▸). The strength of neutron protein crystallography in providing detailed information on H-atom positions is particularly advantageous for the study of CPET in a metalloenzyme in which redox states must be maintained and knowledge of protonation states provides information on proton transfer.

**Figure 6:**
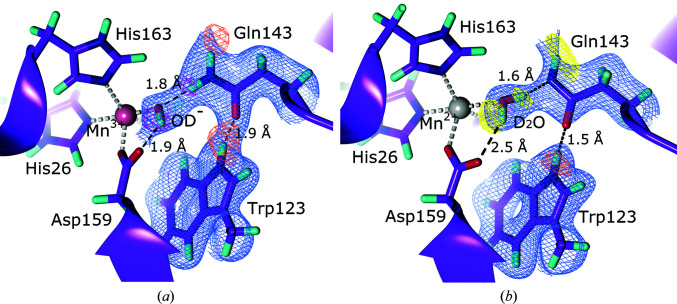
The active site of manganese superoxide dismutase. (*a*) The oxidized active site with a doubly protonated Gln143 (PDB entry 7kks, perdeuterated; Azadmanesh *et al.*, 2021 ▸). 2*F*_o_ − *F*_c_ NSLD map (σ = 1.00) is displayed as a blue mesh; H and D atoms are displayed in white and turquoise, respectively. *F*_o_ − *F*_c_ NSLD omit map is displayed as a mesh with σ = 3.00 in orange and σ = 3.50 in magenta for selected D atoms. (*b*) The reduced active site with a singly protonated Gln143 (PDB entry 7kkw, perdeuterated; Azadmanesh *et al.*, 2021 ▸). 2*F*_o_ − *F*_c_ NSLD density (σ = 1.00) is displayed as a blue mesh; D atoms are displayed in turquoise, respectively. *F*_o_ − *F*_c_ NSLD omit map is displayed as a mesh with σ = 2.50 in yellow and σ = 3.00 in orange for selected D atoms.

To this end, Azadmanesh and coworkers collected room-temperature neutron diffraction data from oxidized Mn(III)SOD and reduced Mn(II)SOD to resolutions of 2.20 and 2.30 Å, respectively (Azadmanesh *et al.*, 2021 ▸). The heavy-atom positions of the MnSOD backbone and side chains were determined using X-ray data sets of the reduced and oxidized forms and these coordinates were used as the starting models for neutron data-only refinement to avoid including X-ray-induced artefacts at the manganese centre. Analysis of the NSLD maps provided unprecedented insight into the protonation states of second-shell residues, particularly His30, Tyr34, Gln143 and Tyr166, while also revealing the role of active-site waters and emphasizing the role of short strong hydrogen bonds (SSHBs) and low-barrier hydrogen bonds (LBHBs) in stabilization during catalysis. The oxidized manganese(III) centre was found to be five-coordinated with a hydroxide (termed WAT1) ligand, confirming earlier DFT calculations (Fig. 6 ▸ *a*; Li *et al.*, 1999 ▸; Han *et al.*, 2002 ▸; Rulíšek & Ryde, 2006 ▸; Kaukonen *et al.*, 2008 ▸). WAT1 was stabilized by hydrogen bonding to Gln143 via its D^ɛ22^ deuteron. Significantly, following reduction to manganese(II), WAT1 was protonated by Gln143 to form water and a deprotonated glutamine present as an amide ion (Fig. 6 ▸ *b*). This finding, although unexpected given a glutamine p*K*_a_ in the range 16–18, can be explained by active-site p*K*_a_ modulation (Eriksson *et al.*, 1995 ▸) as well as by the Trp123 residue that was found to form an SSHB with Gln143, potentially stabilizing the amide form. Furthermore, complementary DFT calculations supported the role of Gln143 as a proton donor to WAT1 as opposed to the previously proposed Tyr34 (Perry *et al.*, 2009 ▸). The role of Tyr34 as a proton donor to WAT1 was further put into question by the finding that in the manganese(III) form Tyr34 is deprotonated, forming an SSHB with a neighbouring WAT2 molecule, ruling out its role as a proton donor upon manganese(III) reduction. One of the active sites of the reduced manganese(II) form revealed a sixth ligand, characterized as a hydroxide from the NSLD maps, in the position opposite to the coordinating Asp159, supporting previous structural findings (Borgstahl *et al.*, 2000 ▸). This hydroxide association is additionally characterized by bond lengthening between manganese(II) and Asp159 and by a reduction in the electropositivity of manganese(II). It is hypothesized that this represents an intermediate prior to WAT1 protonation, since a decrease in the manganese(II) positive charge by hydroxide coordination may result in electronegative polarization of the WAT1 hydroxide, thereby promoting deprotonation of Gln143. Analysis of the NSLD omit maps further revealed unusual protonation states of the active-site channel His30 which is associated with Tyr166.

Both His30 and Tyr166 are also intricately involved in the change in protonation states at the active site. In the oxidized manganese(II) form, Azadmanesh and coworkers observed a doubly deprotonated His30 imidazolate anion, with the nearest D atom refined as being associated with the hydroxyl group of Tyr166. The authors interpret this as Tyr166 interchanging between an ionized and a protonated form that interacts with His30, which alternates in a concerted fashion between a deprotonated imidazolate anion and an N^ɛ2^-protonated form. Although unusual, histidine residues have been observed in an imidazolate form and may play an important role in changes of protonation states during catalysis (Lyubimov *et al.*, 2006 ▸). Upon reduction to the manganese(II) form, His30 was found in an N^δ1^-protonated form with WAT2 having provided the proton, while an LBHB forms between Tyr166 and the N^ɛ2^ atom of His30. These changes in protonation state in the Tyr166–His30 pair can be related to the changes in protonation at the active site via the solvent molecule WAT2, which is involved in the change in protonation states and is replenished from bulk solvent upon catalytic use. The insights gained from analysis of the neutron crystallography structures allowed Azadmanesh and coworkers to propose a reaction mechanism in which changes to metal redox states are coupled to two internal proton transfers as well as two external proton transfers from solvent molecules. The unexpected finding that Gln143 is the WAT1 proton donor is significant for potential future studies of MnSOD upon interaction with substrate or indeed with cryogenically trapped intermediates.

The neutron protein diffraction structures of the oxidized and reduced form of MnSOD provide a wealth of insight into CPET and the important role that second-shell residues have in the modulation of active-site protonation states during catalysis. The neutron structures revealed novel proton donors and unexpected protonation states which may assist in the unravelling of the reaction mechanism in further oxido­reductases in which electron and indeed proton transfers are crucial.

### 2.6. Chlorite dismutase   

**Figure 7:**
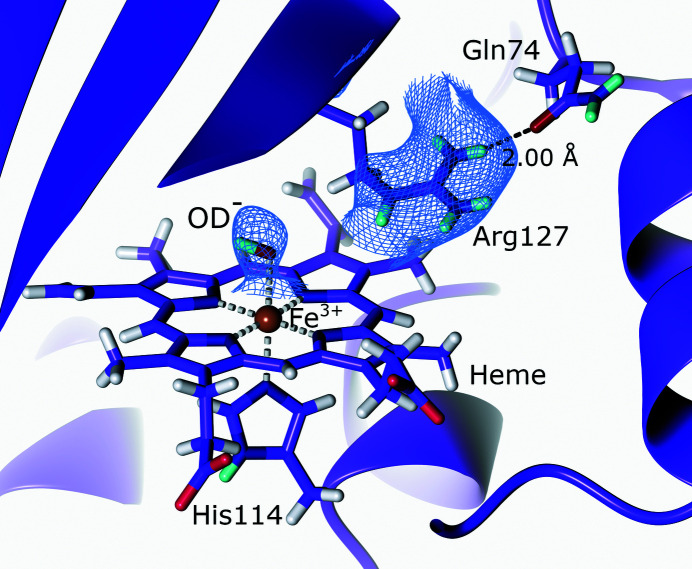
The active site of chlorite dismutase. The active-site Arg127 remains fully protonated in the outward conformation (PDB entry 5nku, H/D exchanged; Schaffner *et al.*, 2017 ▸). 2*F*_o_ − *F*_c_ NSLD map (σ = 1.40) is displayed as a blue mesh; H and D atoms are displayed in white and turquoise, respectively.

The generation of a covalent oxygen–oxygen bond is thus far a biologically rare reaction that has only been attributed to photosystem II and nitric oxide dismutase (Rutherford, 1989 ▸; Ettwig *et al.*, 2010 ▸; Zhu *et al.*, 2019 ▸). A third enzyme, chlorite dismutase, has however garnered much interest for its ability to form an oxygen–oxygen bond during turnover of chlorite (OClO^−^) to chloride (Cl^−^) and molecular dioxygen (O_2_) (Lee *et al.*, 2008 ▸). Chlorite dismutases (Clds) are a family of heme *b*-containing oxidoreductases (EC 1.13.11.49) that are present in prokaryotes (Hofbauer, Schaffner *et al.*, 2014 ▸). Clds can be divided into two clades which differ in their oligomeric organization and subunit structure. Clade 1, the ‘long’ Clds, are present as homopentamers or homohexamers and contain an α-helix-rich N-terminal domain and a heme *b*-containing ferredoxin-like domain (Kumar *et al.*, 2015 ▸; Kostan *et al.*, 2010 ▸). Clade 2, on the other hand, are termed ‘short’ Clds, form homodimers and are shorter than their clade 1 counterparts because they lack the N-terminal α-helix-rich domain while maintaining a similar C-terminal domain (Mlynek *et al.*, 2011 ▸; Celis *et al.*, 2015 ▸). The reaction mechanism of Clds is postulated to follow either a homolytic or a heterolytic route following chlorite binding (Schaffner *et al.*, 2015 ▸). During homolytic cleavage (Keith *et al.*, 2011 ▸) chlorine monoxide (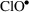) and Compound II are produced, while heterolytic cleavage (Mayfield *et al.*, 2013 ▸) produces hypochlorite (ClO^−^) and Compound I as shown below:
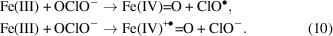



In both cases, following chlorite cleavage there is a rebinding step that produces peroxyhypochlorite (OOCl^−^), which subsequently results in the formation of chloride and dioxygen (Lee *et al.*, 2008 ▸),
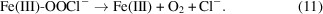



The active-site architecture of Clds includes a proximal histidine that ligates the heme iron, as well as conserved glutamate, lysine and two tryptophan residues (Schaffner *et al.*, 2015 ▸). In the resting state, the distal side of the heme iron is occupied by a water molecule. The only conserved charged residue is an arginine in an otherwise hydrophobic active site. This arginine has been postulated to play a catalytic role and has been found to have an ‘inward’ conformation pointing towards the heme and an ‘outward’ conformation facing towards the substrate-entry channel (Goblirsch *et al.*, 2010 ▸; Kostan *et al.*, 2010 ▸). This flexible arginine residue is believed to be involved in substrate recognition and enzyme stability, as well as playing a role in homolytic/heterolytic cleavage and the subsequent intermediate recombination reaction (Hofbauer, Schaffner *et al.*, 2014 ▸; Schaffner *et al.*, 2015 ▸; Hofbauer, Gruber *et al.*, 2014 ▸). The turnover of Clds shows a strong dependence on pH, a feature that has been linked to the pH-dependent protonation state of the distal arginine residue (Streit *et al.*, 2010 ▸; Blanc *et al.*, 2012 ▸). In order to investigate the role of arginine in Cld catalysis and determine the role of its protonation state in chlorite degradation, Schaffner and coworkers collected a 2.35 Å resolution room-temperature neutron diffraction data set at pH 9.0 as well as a 2.0 Å resolution X-ray diffraction data set from the same crystal for joint refinement for the clade 2 dimeric chlorite dismutase from *Cyanothece* sp. PCC7425 (CCld; Schaffner *et al.*, 2017 ▸). In addition, they collected X-ray data at pH 6.5 and 8.5 and performed stopped-flow, UV–Vis and resonance Raman spectroscopy to determine the role of pH and characterize the reaction mechanism. Analysis of the NSLD maps of the proximal active-site residues at pH 9.0 indicated a rigid hydrogen-bonding network composed of the coordinating His114, Glu167 and Lys92, the hydrogen-bond lengths of which were mostly invariant with change in pH when compared with the X-ray diffraction structures at pH 6.5 and 8.5. On the distal face, the iron was coordinated to a hydroxide (the deprotonated form of water molecule W501) and a second water molecule (W502). The active-site arginine (Arg127) was found to be in the ‘outward’ conformation stabilized by a hydrogen bond to Gln74 (Fig. 7 ▸). Furthermore, Arg127 remained fully protonated at pH 9.0, ruling out its role as a distal base that modulates chlorite degradation during catalysis. These findings agree with studies that found that the guanidinium group of arginine remains charged at high pH in internal protein sites (Harms *et al.*, 2011 ▸; Fitch *et al.*, 2015 ▸).

The structural and spectroscopic findings led the authors to conclude that CCld is most likely to follow a homolytic cleavage pathway with formation of Compound II; however, some Compound I formation observed under acidic conditions means that heterolytic cleavage cannot be unequivocally ruled out. In this mechanism, Arg127 plays a role in substrate recognition in its ‘outward’ conformation and it is possible that it may adopt an ‘inward’ conformation during catalysis by displacing W502 and stabilizing an intermediate. The role of Arg127 in CCld was further examined in mutagenesis studies, which confirmed that its role in the catalytic efficiency of CCld was small (Schmidt *et al.*, 2021 ▸). It was found that disrupting the hydrogen bond from Arg127 to Gln74 by mutating Gln74 to a valine (Q74V) resulted in a more flexible Arg127 that could adopt an ‘inward’ conformation in CCld, while an aspartate mutant (Q74E) locked Arg127 in an ‘outward’ conformation in a salt bridge. It was found that the conformational dynamics of Arg127 has little effect on CCld catalysis since both mutants display a similar catalytic efficiency, which agrees with the neutron diffraction findings. It was found, however, that Arg127 plays a role in the thermal stability of CCld, with the more rigid Q74E mutant showing increased thermal stability. Moreover, Arg127 acts as a gatekeeper to the active site and plays a role in heme coordination of intermediates during catalysis.

A strength of neutron protein diffraction as a probe is the absence of radiation damage and the coincident metal reduction that introduces artefacts in structures solved by X-ray diffraction (Meilleur *et al.*, 2020 ▸; Pfanzagl *et al.*, 2020 ▸). In the neutron diffraction structure, Schaffner and coworkers observed a distance of 2.33 Å between the Fe atom and the hydroxide O atom, a distance that the authors observed to be longer than expected (Schaffner *et al.*, 2017 ▸). Indeed, comparison of several structures indicated a correlation between ligand distance and the reducing power of the radiation used, with distances measured following synchrotron radiation being the longest. The observation of a longer iron–oxygen distance than anticipated could be attributed to the recent finding that chlorite dismutase shows unexpectedly high photosensitivity when exposed to UV–visible light (Mahor *et al.*, 2020 ▸). This observation that even the visible light spectrum induces a degree of photoinactivation in Cld provides a perspective on the care that must be taken when studying photosensitive proteins, since the underlying chemistry may be more dependent on environmental and experimental factors than anticipated.

### 2.7. Amicyanin   

**Figure 8:**
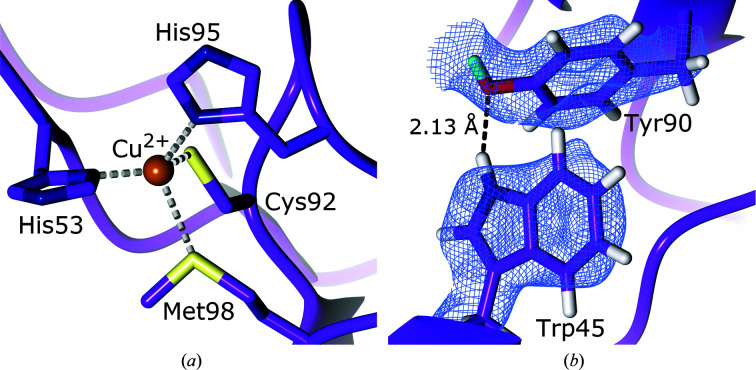
The active site of amicyanin and a strong hydrogen bond to an unexchanged tryptophan (PDB entry 3l45, H/D exchanged; Sukumar *et al.*, 2010 ▸). (*a*) Conserved active-site residues coordinating the copper cofactor. (*b*) The hydrogen bond between the non-exchanged Trp45 and Tyr90. 2*F*_o_ − *F*_c_ NSLD map (σ = 1.00) is displayed as a blue mesh; H and D atoms are displayed in white and turquoise, respectively.

Blue copper proteins are small type I copper proteins that function as electron shuttles (De Rienzo *et al.*, 2000 ▸). Also termed cupredoxins, blue copper proteins function as intermediaries, accepting electrons from one molecule and donating them to another (Sykes, 1991 ▸). Amicyanin (EC 1.4.9.1) is a bacterial cupredoxin of 11.5 kDa containing a copper coordinated to two histidines, a cysteine and a methionine (Fig. 8 ▸ *a*; Choi & Davidson, 2011 ▸). Intermolecular and intramolecular electron transfers play an important role in redox reactions and cellular processes such as respiration and photosynthesis, making an understanding of how proteins control such electron-transfer reactions crucial (Williamson *et al.*, 2014 ▸; Davidson & Jones, 1996 ▸).

In order to investigate the role of protein dynamics in electron transfer, Sukumar and coworkers performed a joint X-ray–neutron study on amicyanin from *Paracoccus denitrificans*, which shuttles electrons from the tryptophan tryptophylquinone (TTQ) cofactor of methylamine dehydrogenase (MADH) to the heme of cytochrome *c*_551i_ (Davidson & Jones, 1996 ▸; Sukumar *et al.*, 2010 ▸). The copper of this amicyanin is coordinated by the imidazole N atoms of His53 and His95 and the S atoms of Cys92 and Met98 (Durley *et al.*, 1993 ▸). All three components of the ternary MADH–amicyanin–cytochrome *c*_551i_ system have been structurally characterized, providing a structural framework for the neutron diffraction studies (Chen *et al.*, 1992 ▸, 1994 ▸; Durley *et al.*, 1993 ▸). An X-ray data set was collected to 1.5 Å resolution and used with a neutron diffraction data set at 1.8 Å resolution for joint X-ray–neutron structure refinement to accurately determine the positions of H/D atoms and the extent of H/D exchange in order to investigate the dynamics and flexibility of amicyanin. Analysis of the electron-density and NSLD maps revealed that seven of 17 buried residues at an intramolecular depth of ≥3.8 Å displayed full H/D exchange, with a valine residue at a depth of 5.5 Å also displaying full H/D exchange. This high level of exchange near the protein core indicates that the protein exhibits a high level of dynamic motion. In contrast, core structural elements of the protein involved in the formation of β-strands did not exchange, confirming their role in protein structural stability. Additionally, 14 residues were found to have non-exchangeable H atoms, including Trp45 and Tyr90, which form a strong hydrogen bond that enhances thermal stability (Fig. 8 ▸ *b*; Dow *et al.*, 2014 ▸). Residues in the vicinity of the copper as well as those involved in mediating electron transfer displayed significant levels of H/D exchange, further highlighting the dynamic nature of amicyanin, while also supporting the role of these residues in long-range electron transfer. These findings shed light on how protein dynamics can regulate electron transfer, and in particular the effect on electronic coupling. Electronic coupling, also termed H_AB_, provides an indication of the likelihood that a reaction will take place when the activation energy is achieved (Williamson *et al.*, 2014 ▸; Cave & Newton, 1997 ▸). Protein dynamics can alter the H_AB_ by decreasing the distance of necessary through-space jumps during electron transfer and increasing the atomic packing density. These alterations would explain the surprisingly low calculated H_AB_ values for the amicyanin crystal structure when compared with the same system in solution (Davidson & Jones, 1996 ▸; Ferrari *et al.*, 2003 ▸). The findings of Sukumar and coworkers illustrate the important role that protein dynamics has in reaction and transfer rates. In addition to examining H/D-exchange levels, Sukumar and coworkers analyzed the presence and orientation of hydrogen bonds within the amicyanin structure. The observed hydrogen bonds in their oxidized amicyanin structure correspond well to those predicted by high-resolution X-ray diffraction structures; however, they observed that the reduced form of amicyanin had five fewer hydrogen bonds, confirming that the reduced state undergoes a conformational change (Zhu *et al.*, 1998 ▸). Analysis of the number and type of hydrogen bonds within 8 Å of the copper indicated a high proportion of C—H⋯*X* bonds, which are weaker than conventional hydrogen bonds. The large number of C—H⋯*X* bonds may function collectively in stabilizing the structure of amicyanin (Iwaoka, 2015 ▸). The joint X-ray/neutron diffraction study presented by Sukumar and coworkers provides insight into the capability of neutron diffraction to study H/D-exchange patterns to inform on protein dynamics and also to reveal the importance of conventional and nonconventional hydrogen bonds in the protein structure.

### 2.8. Manganese catalase   

**Figure 9:**
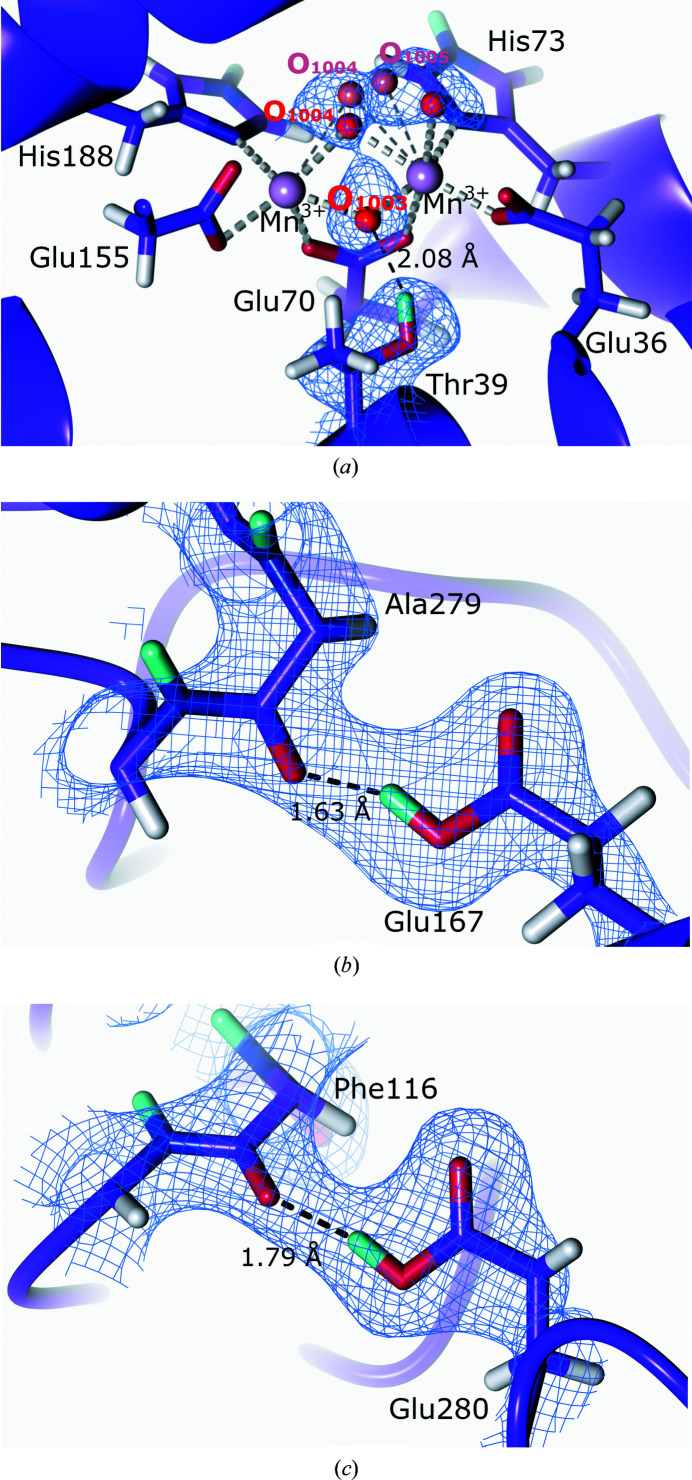
The active site and protonation states of glutamate residues with backbone carbonyl O atoms (PDB entry 6kk8, H/D exchanged; Yamada *et al.*, 2019 ▸). 2*F*_o_ − *F*_c_ NSLD map (σ = 1.00) is displayed as a blue mesh; H and D atoms are displayed in white and turquoise, respectively. (*a*) Manganese catalase active site with the bridging O atoms coordinated to the two manganese cofactors. Bridging atoms O_1004_ and O_1005_ have alternate conformations, which are shown in red and pale red. (*b*) Single protonation of Glu167 forming a hydrogen bond to the Ala279 carbonyl group oxygen. (*c*) Single protonation of Glu280 forming a hydrogen bond to the Phe116 carbonyl group oxygen.

Hydrogen peroxide (H_2_O_2_) serves various functions in cellular metabolism, functioning as a signalling molecule and a cellular pathway regulator; however, this cytotoxic molecule can also be produced as a byproduct of aerobic metabolism, requiring rapid removal (Lennicke *et al.*, 2015 ▸; Gough & Cotter, 2011 ▸; Fang, 2004 ▸). Catalases are metalloproteins that are present in microbes, plants and animals and function to remove H_2_O_2_ before it results in potential oxidative cellular damage by degrading it into water and molecular oxygen (Glorieux & Calderon, 2017 ▸). There are two families of catalases with distinct cofactors, structures and chemistry: heme and nonheme catalases (Chelikani *et al.*, 2004 ▸). Heme catalases contain an iron porphyrin cofactor and degrade H_2_O_2_ *via* a Compound I intermediate (Zámocký & Koller, 1999 ▸). Nonheme catalases, also known as manganese catalases (MnCat; EC 1.11.1.6), have been found in bacteria and archaea (Crichton, 2019 ▸). Structural studies revealed that MnCat is a globular protein consisting of 30 kDa monomers forming a homohexameric structure (Antonyuk *et al.*, 2000 ▸; Barynin *et al.*, 2001 ▸). The active site contains two manganese ions connected by two solvent-derived μ-oxo bridging molecules and a glutamic acid carboxyl group. The first coordination shell of this bimetallic core is completed by two glutamic acid residues and two histidine residues. The overall reaction of MnCat involves oxidative and reductive half reactions to give a net reaction in which two H_2_O_2_ molecules produce two molecules of water and molecular oxygen as shown below (Whittaker, 2012 ▸):
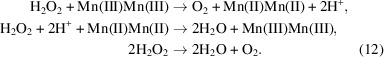



In its resting state, MnCat is present as oxidized Mn(III)Mn(III), which is reduced to Mn(II)Mn(II) during catalysis; however, the addition of oxidizing or reducing agents can also result in mixed-valence states such as Mn(II)Mn(III) and Mn(III)Mn(IV) (Kono & Fridovich, 1983 ▸; Waldo *et al.*, 1991 ▸; Waldo & Penner-Hahn, 1995 ▸). During the MnCat reaction mechanism, the active-site manganese molecules undergo changes in redox states and act as electron repositories in a reaction cycle that contains multiple intermediates with varied protonation states (Crichton, 2019 ▸). The identity of the bridging ligands during catalysis remains to be established; however, it has been proposed that in the resting Mn(II)Mn(III) state the active site contains a μ-oxo and a μ-OH^−^ bridge, which converts to Mn(II)Mn(II) with a μ-OH^−^ and a μ-H_2_O bridge following H_2_O_2_ binding and oxidation (Boelrijk & Dismukes, 2000 ▸). To structurally investigate the protonation states in the active site of the thermostable MnCat from *Thermus thermophilus*, Yamada and coworkers collected a 2.35 Å resolution neutron diffraction data set as well as a 1.37 Å resolution X-ray diffraction data set from the same crystal for joint refinement (Yamada *et al.*, 2019 ▸). Crystallization was performed under basic conditions to ensure the Mn(III)Mn(III) oxidation state. Analysis of the NSLD maps of the active site confirmed the presence of the glutamate bridging ligand as well as a di-μ-oxodimanganese cluster (Fig. 9 ▸ *a*). The μ-bridging O atom closest to the μ-bridging Glu70 was found to be deprotonated in the μ-oxo form and formed a hydrogen bond to Thr39. However, as had also been observed in an earlier 1 Å resolution X-ray structure, a further active-site bridging O atom was disordered, which complicated the interpretation of the protonation state of the 2.35 Å resolution neutron structure (Antonyuk *et al.*, 2000 ▸).

The neutron diffraction structure also indicated protonation of Glu167 and Glu280, which are present at the hexamer boundaries, enabling them to form hydrogen bonds (Figs. 9 ▸ *b* and 9 ▸ *c*). The protonated state of these glutamic acid residues under the basic crystallization conditions can be rationalized by the tight packing of the hexamer, effectively barricading these residues. While this neutron diffraction study did not allow complete interpretation of the bridging ligands in the active site, it represents an exciting first insight into the resting state of MnCat. The reaction mechanism of MnCat comprises several changes of protonation and oxidation states, which are ideal for study using neutron protein diffraction. Future studies following the binding of the H_2_O_2_ substrate or the addition of reductants opens the possibility of further mechanistic findings.

## 3. Perspective   

Visualization of H atoms during proton transfer, changes in protonation state and hydrogen-bond network rearrangement is central to determining the reaction mechanism of oxido­reductases (Fontecilla-Camps & Nicolet, 2014 ▸; Stephanos & Addison, 2014 ▸). A further important consideration in the oxidoreductase mechanism is the delivery of electrons to drive catalysis. Electron delivery often occurs via a reductase partner protein through inter-protein electron transfer (Antonyuk *et al.*, 2013 ▸). Association with the associated reductase often results in conformational changes and regulation of activity similar to the modulation by second-shell residues, as has been demonstrated for cytochrome P450cam in interaction with putidaredoxin (Pdx; Liou *et al.*, 2016 ▸). Small-angle neutron scattering (SANS) allows the association of the metalloprotein with its partner protein to be probed, providing the advantage of contrast matching through selective deuteration to highlight changes in only the component of interest while not inducing radiation damage (Ashkar *et al.*, 2018 ▸). Recent advances in these interactions have been made by SANS on the interactions of sulfite reductase (SiR; Tavolieri *et al.*, 2019 ▸), cytochrome P450 reductase (CPR; Freeman *et al.*, 2018 ▸) and cellobiose dehydrogenase (CDH; Bodenheimer *et al.*, 2017 ▸, 2018 ▸). These SANS studies serve to further expand the insights gained into metalloproteins, thereby providing a more complete mechanistic picture. Neutron protein crystallography serves as a valuable complement to further structural techniques for the elucidation of protein chemistry. As the flux of neutron sources and the ability to cryo-trap intermediates in the protein reaction mechanism improve, our understanding of metalloprotein chemistry at an atomic level will greatly advance.

## Figures and Tables

**Table 1:** Neutron protein crystallography structures of transition-state metal oxidoreductases

Protein	PDB entry	Sequence length	Metal cofactor	Conditions	Deuteration level	Temperature (K)	Neutron resolution (Å)	X-ray resolution (Å)	Space group	*a*, *b*, *c* (Å)
Copper nitrite reductase (*Achromobacter cycloclastes*)	6gtj	340	Cu^2+^	Resting state, pD 5.4	Perdeuterated	293	1.80	N/A	*P*2_1_3	97.98, 97.98, 97.98
Copper nitrite reductase (*Geobacillus thermodenitrificans*)	6l46	323	Cu^2+^	Resting state, pD 5.3	H/D exchanged	100	1.50	1.30	*H*3	114.21, 114.21, 83.69
Copper amine oxidase (*Arthrobacter globiformis*)	6l9c	621	Cu^2+^	Cofactor TPQ bound	H/D exchanged	100	1.72	1.14	*C*121	157.55, 61.78, 92.33
Ferric cytochrome *c* peroxidase (*Saccharomyces cerevisiae*)	4cvi	294	Fe^3+^	Ferric heme	H/D exchanged	290	2.41	2.10	*P*2_1_2_1_2_1_	51.70, 76.80, 107.60
Ferric cytochrome *c* peroxidase (*Saccharomyces cerevisiae*)	4cvj	294	Fe^4+^	Compound I heme	H/D exchanged	100	2.50	2.18	*P*2_1_2_1_2_1_	51.19, 75.83, 107.59
Ferric ascorbate peroxidase (*Glycine max*)	5jpr	261	Fe^4+^	Compound II heme	H/D exchanged	100	2.20	1.81	*P*4_2_2_1_2	82.10, 82.10, 75.16
Ferric ascorbate peroxidase (*Glycine max*)	6tae	261	Fe^3+^	Ferric heme	Perdeuterated	100	2.22	1.90	*P*4_2_2_1_2	82.90, 82.90, 75.87
Ferric ascorbate peroxidase (*Glycine max*)	6xv4	261	Fe^3+^	Co-substrate ascorbic acid bound	Perdeuterated	100	2.09	1.90	*P*4_2_2_1_2	81.86, 81.86, 74.97
Lytic polysaccharide monooxygenase (*Neurospora crassa*)	5tki	223	Cu^2+^	Resting state	H/D exchanged	298	2.12	1.50	*P*12_1_1	68.12, 42.23, 70.29
Lytic polysaccharide monooxygenase (*Jonesia denitrificans*)	5vg1	142	Cu^2+^	Dioxygen species	H/D exchanged	295	2.10	N/A	*P*2_1_2_1_2_1_	32.50, 76.40, 122.10
Superoxide dismutase (*Homo sapiens*)	7kks, 7kku	199	Mn^3+^	Oxidized	Perdeuterated	296	2.20	2.02	*P*6_1_22	81.30, 81.30, 241.84
Superoxide dismutase (*Homo sapiens*)	7kkw, 7klb	199	Mn^2+^	Reduced	Perdeuterated	296	2.30	2.16	*P*6_1_22	81.33, 81.33, 242.88
Chlorite dismutase (*Cyanothece* PCC7425)	5nku	188	Fe^3+^	Ferric heme, pD 9.4	H/D exchanged	293	2.35	2.00	*P*1	52.43, 53.02, 55.34
Amicyanin (*Paracoccus denitrificans*)	3l45	105	Cu^2+^	Resting state	H/D exchanged	293	1.80	1.50	*P*12_1_1	27.54, 56.58, 28.86
Manganese catalase (*Thermus thermophilus* HB27)	6kk8	302	Mn^3+^	Resting state	H/D exchanged	293	2.35	1.37	*P*2_1_3	133.40, 133.40, 133.40
